# Quality Assessment in Supportive Care in Head and Neck Cancer

**DOI:** 10.3389/fonc.2019.00926

**Published:** 2019-09-18

**Authors:** Pierluigi Bonomo, Alberto Paderno, Davide Mattavelli, Sadamoto Zenda, Stefano Cavalieri, Paolo Bossi

**Affiliations:** ^1^Department of Radiation Oncology, Azienda Ospedaliero–Universitaria Careggi, University of Florence, Florence, Italy; ^2^Unit of Otorhinolaryngology, Department of Surgical Specialties, ASST Spedali Civili di Brescia, Brescia, Italy; ^3^Department of Radiation Oncology, National Cancer Center Hospital East, Kashiwa, Japan; ^4^Head and Neck Medical Oncology Unit, Fondazione IRCCS Istituto Nazionale dei Tumori di Milano, Milan, Italy; ^5^Medical Oncology Unit, Department of Medical Oncology, ASST Spedali Civili di Brescia, Brescia, Italy; ^6^Department of Medical and Surgical Specialties, Radiological Sciences and Public Health, University of Brescia, Brescia, Italy

**Keywords:** supportive care, head and neck cancer, quality assessment, multimodal treatment, surgery, chemotherapy, radiotherapy

## Abstract

Quality assessment is a key issue in every clinical intervention, to be periodically performed so to measure the adherence to standard and to possibly implement strategies to improve its performance. This topic is rarely discussed for what concerns supportive care; however, it is necessary to verify the quality of the supportive measures; “supportive care makes excellent cancer care possible,” as stated by the Multinational Association of Supportive Care in Cancer (MASCC). In this regard, the quality of supportive care in head and neck cancer patients is a crucial topic, both to allow administration of treatments according to planned dose intensity or surgical indications and to maintain or improve patients' quality of life. This paper aims to provide insight on state of the art supportive care and its future developments for locally advanced and recurrent/metastatic head and neck cancer, with a focus on quality assessment in relation to surgery, radiotherapy, and systemic therapy.

## Defining the Context: Why Supportive Care Is Necessary in Head and Neck Cancer?

The diagnosis of head and neck cancer (HNC) represents one of the most challenging scenarios in oncology, which both the affected patient and the treating physician have to deal with. To a variable extent, throughout its natural history, the progression of HNC is associated with an increasingly heavier burden of symptoms, altering the ability to eat, drink, swallow, speak, and breathe normally. Intrinsically, HNC may be the cause of severe pain (1), significant reduction of dietary intake (2), uncontrolled bleeding (3), disfigurement (4), psychological distress (5), social retirement (6), and overall marked impairment in quality of life (7). Moreover, the extent of symptoms induced by the disease may have a detrimental effect on survival. In view of the biological aggressiveness of HNC at a loco-regional level, symptom control is one of the key treatment goals pursued both in the curative and palliative setting, taking also into account that many patients consider it as their top priority even over survival (8–10).

Multimodal management of HNC is frequently associated with prohibitive toxicity. In ensuing randomized phase III trials where cisplatin-radiotherapy (RT) combination was the treatment backbone in control arms, severe toxicity rates ranged between 81.7 and 87.6% (11–13). Surgical management of HNC is also complex, with post-operative complications yielding a 19.4% readmission rate within 30 days of reconstruction surgery in referral centers (14). For recurrent and/or metastatic (RM) disease, first-line standard of care (cetuximab combined with platinum−5-fluorouracil doublet) is associated with substantial toxicity (82% incidence of grade 3/4 adverse events) (15). More recently, the Keynote-048 clinical trial showed the efficacy of anti-PD1 (programmed death protein 1) pembrolizumab both as monotherapy and in addition to cisplatin-5-fluorouracil doublet (16); therefore, supportive care should focus also on the management of immune-related adverse events, such as endocrinopathies (e.g., hypothyroidism, hypophysitis), liver toxicity, and diarrhea.

**Figure 1:**
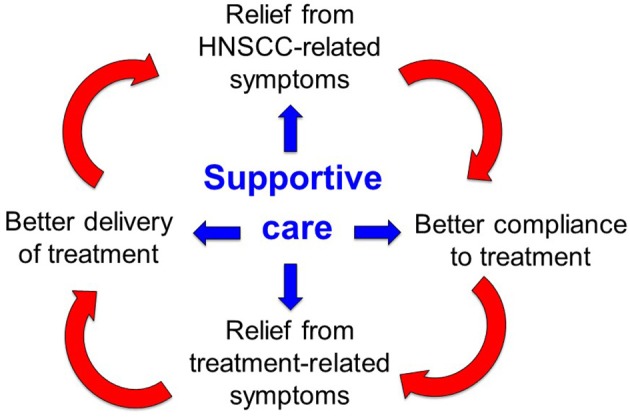
Virtuous cycle of the supportive care in head and neck cancer.

Given these premises, supportive care is of paramount importance along the whole disease trajectory of HNC: it entails all the pharmacological interventions and domain-specific processes aimed to prevent, manage, and mitigate the multifactorial burden of symptoms that may occur as a consequence of the disease and/or its treatments (Figure 1). The timely implementation of intensive supportive care is crucial for oncologic success in patients with head and neck cancer. In this perspective, a virtuous circle can be envisaged (Figure 1): ensuring that patients receive the intended treatment intensity is of utmost importance for HNC outcome: delivering >200 mg/m^2^ cisplatin dose (17), avoiding RT breaks (18), keeping a time interval <50 days between surgery and RT start (19) and achieving a prolonged treatment duration with maintenance cetuximab (20) are such known examples. In addition, addressing the acute side effects induced by multimodal treatment with adequate supportive care may be extremely relevant in order to prevent or mitigate the transition to late consequential toxicity (21). Many HNC survivors are burdened with long-lasting symptoms inflicting on their quality of life and global functioning (22), ultimately leading to potential non-cancer-related (intercurrent) mortality (23). How to assess the quality of supportive care received by the patients throughout their disease trajectory, how to control for its application and how to capture its potential impact on treatment outcome are unmet needs in head and neck oncology. The complexity of care for HNC is reflected by the notion that being treated at low-volume centers may be detrimental to survival (24, 25), underlining the importance of multidisciplinary expertise in treating the disease but also of other factors, such as the prompt availability of multidimensional supportive care.

This paper aims to provide insight on state of the art supportive care and its future developments for locally advanced and RM HNC, with a focus on quality assessment in relation to surgery, RT, and systemic therapy (Table 1).

**Table 1:** Main issues in supportive care for HNC patients and proposed quality metrics.

**Treatment**	**Supportive care issues**	**Quality metrics**
Surgery	Prevention of SSI	Presence of evidence-based guidelines on antibiotic-prophylaxis based on prevalence of SSI and resistances to antibiotics
	Perioperative pain management	Standardized assessment of pain and its characteristics for every pt Pre, intra, and postoperative analgesic protocols
	Nutritional rehabilitation after surgery on the upper aerodigestive tract	Rate of pts with oral diet within the 5th postoperative day
Radio(chemo)therapy	Nutritional assessment before and during radio(chemo)therapy	Rate of pts receiving validated nutritional screening tools (e.g., NRS-2002, MNA, MST, MUST)
	Nutritional enteral/parenteral support	Adherence to International guidelines (e.g., ESPEN guidelines)
	Prevention of swallowing problems related to RT	Presence of a swallowing program Involvement of physiatrists/speech therapist in the MDT
	Treatment of RT-induced pain	Continuous assessment of pain during RT Protocol of treatment for background and breakthrough cancer pain
	Prevention and treatment of mucositis	Adherence to international guidelines (e.g., MASCC guidelines)
	Prevention of major infections during chemotherapy and/or RT	Rate of major infections during treatments Knowledge about pathogenic microorganisms and patterns of antibiotic resistance
	Psychological distress during treatment	Rate of pts receiving screening for distress Involvement of psycho-oncologists in the MDT (as needed)
Palliative care	Early approach with simultaneous care in the RM phase of disease	Quality of life and pt's satisfaction Rate of unplanned access to emergency services Rate of pts dying in hospice or with a home care
	Avoiding active oncological treatments in the end-of-life period	Rate of pts receiving a new treatment in the last 3 months of life Rate of pts receiving any systemic treatment in the last month of life

*SSI, surgical site infections; Pt, patient; RT, radiation; MDT, multidisciplinary team; MASCC, Multinational Association of Supportive Care in Cancer; RM, recurrent and/or metastatic*.

## Quality of Supportive Care in Surgery

### Prevention of Infections and Methods for Evaluating Their Application

Surgical site infection (SSI) is a relatively frequent complication that may follow any type of surgical procedure, potentially resulting in delayed wound healing, wound breakdown, fistula formation, and compromised tissue reconstruction. Various organizations have developed guidelines detailing evidence-based criteria aimed to minimize this issue in different surgical specialties (26, 27). However, while commonly accepted antiseptic interventions represent the mainstay of surgery, head, and neck surgical procedures may need specific considerations on some of these concepts (28). A first consideration is that, in order to optimize outcomes and improve data collection, surgical patients should be assessed in a standardized manner: adequately classifying wound type (i.e., World Health Organization classification), determining risk factors for SSI, applying a predetermined protocol of antibiotic prophylaxis (AP), and assessing SSI according to accepted grading scales (29, 30).

Secondly, it should be underlined that AP is still a widely debated issue in head and neck surgery. In spite of growing evidence on its ideal duration, there is a lack of high-quality data concerning the choice of antibiotic type. Furthermore, AP is frequently administered on the basis of local indications or surgeon's personal choice, without relying on sound evidence-based criteria.

Considering available data, clean surgical procedures (e.g., thyroidectomy, parotidectomy, and submandibular gland excision) do not routinely require AP, since SSI occur in <1% of patients (31). However, the upper aerodigestive tract mucosal lining is often disrupted during head and neck surgery, resulting into a “clean-contaminated field.” In this setting, a series of randomized trials clearly established the need for AP (32–34). Both randomized trials and retrospective reviews showed no additional benefits for a duration of AP longer than 24 h (35–39). In fact, prolonged courses of AP did not improve protection against SSI and had a higher incidence of antibiotic-related complications. In this field, antimicrobial stewardship programs play a pivotal role in monitoring and improvement of antimicrobial use and patient outcome (40, 41), granting the use of an appropriate antibiotic spectrum, dosage, and duration needed to prevent or treat infection, thus decreasing the use of extended spectrum agents. In fact, selection of antibiotics is influenced by regional policies, availability, and resistances. Ideally, each Institution should evaluate prevalence of SSI and distribution of resistances to provide evidence-based guidelines on the best AP in each setting.

### Pain Control After Surgery: Guidelines and Application

Widely accepted guidelines and indications on postoperative pain (PP) control in head and neck surgery are lacking. However, it should be noted that effective management of acute PP reduces morbidity, hospitalizations, and hospital costs, while increasing patient satisfaction (42). On the other hand, narcotic medication regimens commonly used to treat PP are associated with constipation, nausea, and long-term addiction (43, 44). Consequently, the main objectives of pain management approaches are to provide an optimal PP control, while reducing the need or dose of opioids, and minimizing drug-related sequelae/side effects. In order to meet these requirements, the type of surgery should be classified according to its related pain levels (45). This allows to apply a standardized perioperative pain management protocol encompassing the pre-, intra-, and post-operative phases, aimed at maximizing efficacy while minimizing opioid use. In fact, preventive analgesia can decrease central sensitization and hyperalgesia (46), leading to a significant reduction in PP medication requirements (47). In particular, available data on otolaryngology does not show a significant increase in the risk of postoperative bleeding using NSAIDs (48), justifying their routine use before shifting to opioids. Adjunctively, local and regional intraoperative anesthesia proved to reduce analgesic consumption in the postoperative period without any increase in PP (47).

Pain should be constantly assessed using standardized scales, such as numerical rating scale or visual analog scale. Pain characteristics (background, breakthrough, and swallowing-related pain) ought to be recorded and detailed as well, in order to better tailor treatments (49).

### Pre-habilitation in Surgery: the ERAS Protocol Example

The Enhanced Recovery After Surgery (ERAS) protocol represents a multimodal and multidisciplinary approach to surgical patients aimed to enhance the quality of recovery after surgery. ERAS program includes different items encompassing preoperative patient preparation, reduction of stress response to surgery, prevention of complications, and rapid return to normal functions. Cooperation of different specialists, patient collaboration, and continuous internal audit to improve the adherence to the protocol are key-points to success.

The experience in HNC is very limited, although critical issues specific to head and neck patients (cancer-related malnourishment, high comorbidity burden due to smoke and alcohol, postoperative swallowing rehabilitation, tracheostomy) may negatively impact the risk of complications and the length of hospitalization.

In 2017, an international expert group in collaboration with ERAS Society published a consensus protocol on the optimal care of patients undergoing major head and neck surgery (50); these recommendations represents the “state-of-the-art” guidelines to implement an ERAS protocol in HNC (Table 2).

**Table 2:** The most relevant domains for ERAS protocol in head and neck cancer patients.

**Preadmission education, aimed at preparing both the patient and the family to the expected recovery course**
Preoperative nutritional evaluation, and implementation of a nutritional plan to correct a malnourishment status (with possible placement of a nasogastric tube, or gastrostomy tube)
Reduction of preoperative fasting and administration of a carbohydrate-enriched drink to reduce catabolism and insulin resistance
Thromboembolic and antibiotic prophylaxis
Correct anesthesiologic management, which includes prevention of hypothermia and adequate perioperative fluid load (near zero balance, or goal-directed fluid therapy)
Postoperative nausea and vomit prophylaxis, and pain management
Mobilization within the first 24 h and postoperative pulmonary physical therapy
Early postoperative nutrition (within 24 h) and early oral feeding
Restricted indications to tracheotomy and timely decannulation with surgical closure, which can speed up swallowing recovery and shorten hospitalization

The most relevant and controversial aspect of a rapid rehabilitation in head and neck is probably early oral feeding after surgery on the pharyngolaryngeal axis. A systematic review including four cohort studies and four randomized clinical trials demonstrated that an early and gradual reintroduction of an oral diet (between the 2nd and 5th postoperative day) after total (pharyngo)laryngectomy is not associated with an increased risk of salivary fistula (51). Conversely, it shortens the hospital stay with a possible positive impact on patients' psychological status and costs (52).

Overall, only a few papers regarding the implementation of an ERAS program in head and neck cancer have been published so far. Imai et al. compared 28 patients treated in accordance to an ERAS protocol to an historical control group and demonstrated a reduction in complication rate (17.9 vs. 36.7%; *p* = 0.07), while no difference was found according to the hospitalization time (53). Conversely, Bater et al. showed a relevant shortening in hospitalization (10 vs. 14 days; *p* = 0.003), while no difference in complication and readmission rates was evident (54). Surprisingly, McMahon et al. found no advantage for ERAS program in any outcome (55); however, they did not report any data on compliance to the protocol, which is widely recognized as one of the major factor to improve recovery outcomes (56). Overall, evidence-based data to assess the degree of benefit of an ERAS protocol in head and neck surgery is currently lacking, and large, high-quality studies are warranted. Another relevant pending question is the possible delay of adjuvant treatments due to early rehabilitation protocols. So far, the implementation of an ERAS protocol has never shown any increase of complication rate, or hospitalization length that could support this risk; however, this aspect should be considered as a relevant quality indicator in future studies.

## Quality Assessment in Supportive Care in Radiation With or Without Systemic Therapy

RT and chemoradiotherapy (CRT) are widely used in patients with locally advanced HNSCC, both as curative and postoperative approaches. There is a link between the compliance with the programmed therapeutic plan and outcome; this association can be preserved through the implementation of an integrated supportive care plan, declined into its nutritional, swallowing, exercise, psychological, and symptom control dimensions. Therefore, compliance to the treatment can mirror the quality of the supportive care implemented in the patient's pathway.

### Nutritional Support

Weight loss during RT/CRT is associated with a significant toxicity, especially in terms of mucositis, often leading to malnutrition. In some cases, weight loss-induced body shape change leads to the necessity of re-planning of RT plan (57, 58).

Nutritional screening assessment at baseline is paramount to better frame the actual needs of each patient, in order to provide prompt interventions. After screening, periodical nutritional assessments are strongly recommended during CRT as well (59). As in every field of HNC patients' management, also for nutritional support a multidisciplinary approach is essential to tailor patients' needs and to address specific therapeutic strategies. To provide evidence-based standard of care while prescribing enteral and parenteral nutrition, adherence to international guidelines is strongly recommended (60) and adherence to guidelines should be considered a way to assess quality of the center.

### Swallowing Exercises

In HNC patients, dysphagia is an important treatment-related side effect in patients treated with RT or CRT. This symptom can lead to severe life-threating complications, such as aspiration pneumonitis and malnutrition, and a feeding tube is often needed.

Several reports showed swallowing exercise may improve dysphagia and quality of life (61–65). However, since adherence to behavioral intervention may vary among patients, again a multidisciplinary approach is strongly encouraged (66). Indeed, the involvement of physiatrists and speech therapists could provide a precious help in keeping a better compliance and in avoiding both early and late complications. In this regard, quality assessment of prevention and cure of this symptom should be performed considering whether the center has implemented a swallowing program and whether a multidisciplinary group is involved.

### Pain Therapy

Radiation-induced mucositis causes severe pain and poor oral intake, and often results in unplanned treatment breaks, clinic visits, and hospitalizations.

Pain during RT usually worsen in the second half of treatment period, then improve 1–2 weeks after the conclusion of RT/CRT. Risk of treatment-related pain depends mainly on the distribution of RT dose on organs at risk (67).

Local approaches to prevent oral mucositis should be encouraged. In particular, an adequate oral hygiene and sodium bicarbonate oral rinses should be started since the beginning of treatment (68, 69). Whenever the pain of focal sites of mucositis are not controlled by treatments, the topical application of lidocaine can improve the symptom greatly, even if for a limited time period (49). To prevent painful radiodermatitis, there is strong evidence supporting the efficacy of gentle washing and moistening of the wound healing environment (70–72).

A thorough pain control program should include an early detection of the symptom and a prompt start of major analgesic therapy, generally overtaking the traditional three steps proposed by the World Health Organization (WHO). Indeed, to control odynophagia strong opioids (e.g., oral morphine sulfate) should be started precociously on an around-the-clock basis, especially before meals (73). Then, in case of background pain not manageable with dose escalation, a prolonged-release strong opioid should be started. In this regard, the use of opioids as pain treatment, tailoring the treatment according to background and breakthrough pain could be considered as metrics of the quality in head and neck cancer care.

Physicians expert in pain therapy should be involved in case of pain not manageable with pharmacological and non-pharmacological approaches.

#### Infections

HNC patients are known to be generally immunosuppressed (74). In this setting, the potential harm of treatment-induced further immunosuppression plays a definite role in determining a higher risk of infections. As previously mentioned, the prevention of oral and oropharyngeal mucositis and neck radiodermatitis through local approaches is crucial. Indeed, the radiation-induced solution of continuity of the natural integrity of the anatomical barrier made of anatomically intact mucosa or skin can be an entrance gate for infections. Moreover, chemotherapy-induced neutropenia may impair further the ability to fight against infections. In addition, some medical devices like central intravenous catheters could be a further significant risk factors for systemic infections.

For these reasons, an accurate follow-up with acute phase reactants (e.g., C-reactive protein; procalcitonin in case of bacterial infections) should be performed, especially in patients receiving chemotherapy. Indeed, fever may not be observed due to the anergy of head and neck cancer patients.

Sepsis is defined as life-threatening organ dysfunction caused by a dysregulated host response to infection, whereas septic shock is a subset of sepsis in which underlying circulatory and cellular/metabolic abnormalities are profound enough to substantially increase mortality (75). These definitions should be always kept in mind while approaching a HNC patient experiencing a systemic infection during RT/CRT. In case of acute infections in frail patients, antimicrobial drugs should be promptly started, taking into account the specific epidemiology of the geographic area.

Therefore, quality in preventing and treating infections in HNC patients can be measured by the rate of major infections during treatment and by the knowledge about the most frequent microbiological causes of infection, as well as the pattern of antibiotic resistance typical of that area.

### Availability of Psychological Consultation

Between 22 and 35% of RT outpatients report clinically relevant psychological distress (76–80) and they often negatively influence treatment compliance.

Distress screening for all patients receiving RT is recommended and patients' wish for psychological support should be detected. Both patients and their caregivers should be psychologically assessed, and these evaluations should be carried on constantly during treatments (81). This is the reason why psycho-oncologists should be involved in multidisciplinary HNC boards.

One of the most commonly used distress screening questionnaires is the National Comprehensive Cancer Network (NCCN) Distress Thermometer, sometimes administered with its modifiable Problem Checklist (82). It is advisable that quality assessments of the psychological support offered to patients are regularly carried out. Possible indicators are the rate of admitted/screened patients, the adherence of the patient to the agreed schedule and his/her satisfaction that could be investigated thanks to dedicated questionnaires.

## Quality Assessment in Supportive Care During Treatment for Recurrent and/or Metastatic Disease

### The Concept of Simultaneous Care to Allow for Better Patient Care

Patients with RM HNC suffer of physical, emotional, and functional symptoms, which greatly impact on their quality of life. Symptoms often affect vital functions such as eating, talking, and breathing. The facial aspect is often altered, as well as taste, hearing, and swallowing. Moreover, compared to other cancer sites, HNC patients have the highest intensity of pain (83). These aspects suggest the need of high levels of palliative and supportive care both for the patients and their family caregivers. In this regard, we need a defined framework to provide supportive and palliative care which can be directly embedded in the trajectory of care of RM HNC patients. Multimodal multidisciplinary interventions are essential for RM patients, including for instance nutritional, pain, psychological aspects, as well as targeting functional issues (84).

From this point of view, RM HNC patients are candidates for high levels of palliative and supportive care interventions from the earliest stages of diagnosis.

Indeed, a meta-analysis showed that early palliative care is able to improve patients' quality of life (85). Therefore, this precious support should not be considered only in the last months of life.

In oncology, the early onset of a palliative and supportive care in oncology patients treatment showed to favorably impact on patients' quality of life, perception of disease, and also on end-of-life choices; more controversial is the beneficial effect on OS (86).

Recently, a study in brain cancers, a setting of care sharing challenges of physical, psychological, and functional issues with HNC patients, tried to set guidelines for supportive/palliative care (87). Similarly, there is a need to set a defined framework for the early introduction of supportive care in RM HNC patients, involving family members and caregivers as well as healthcare professionals. The compliance with this feasible framework could represent one element to evaluate how the patient is cared for.

In this regard, the assessment of the quality of supportive care during the RM phase of disease is extremely important to improve the treatment of these very frail patients. Metrics of evaluation could be represented by the number of unplanned accesses to emergency services or unplanned visits to oncology department, by the quality of life reported by the patients and caregivers and by the more controversial issue of patient's awareness about prognosis.

Another quality metric, even if difficult to be objectively measured, is the ability of the multidisciplinary team following RM HNC patients to anticipate and address emergency symptoms, such as airway obstruction and bleeding.

### Discussing End of Life Choices: the Importance to Make It Early

The conventional model of shared decision-making has been shown not to fit with HNC patients suffering from pain, discomfort, and fear of imminent death (88). Often, they rely on trust and confidence with the physician, accepting treatments in the hope of “doing something” against the disease.

Therefore, anticipating the discussions about choices regarding nutrition and breathing problems, type of pain therapy, and intensity of active oncological treatment is essential to define a shared pathway of care, which could also take the patient's preferences into considerations.

The trade-off between quantity and quality of life is the crucial point in the approach to RM patients, particularly after failing a first-line treatment. An open discussion should incorporate the topics of prognosis, incremental benefit expected by a new treatment, possible complications induced by the disease and by the therapies themselves. Incorporating the results of this discussion into the patient's chart should be considered as one of the tasks of the check list of patients presenting with RM disease. Periodic re-evaluation of these choices is necessary, as patients expectations and desires may change during time.

### Quality Assessment: Chemotherapy in the End of Life Period

Avoiding to perform chemotherapy in the last month (or 14 days) of life is one of the point any oncologist should consider to improve patient's quality of life and to perform an open discussion about the end of life choices (89–91). Prolonged administration of chemotherapy when clinical conditions are worsening is often a waste of time and quality of life for the patient, with an increase of toxicities, admission to emergency room and unnecessary exams and hospital visits. Continuous assessment of patients who died from cancer receiving chemotherapy in the last period of life has been considered as a key quality measure. A low-value care for patients in this setting is defined as any treatment not impacting on survival and not improving quality of life (92).

An early involvement of the supportive and palliative team is a central issue to allow better patient information and care and to avoid administering chemotherapy in the last period of life (93, 94).

## Conclusions

The topic of quality assessment is rarely discussed for what concerns supportive care; however, it is necessary to verify the quality of the supportive measures because “supportive care makes excellent cancer care possible,” as it is stated by the Multinational Association of Supportive Care in Cancer (MASCC). In this regard, next step to implement supportive care in HNC should be the creation of checklists specific to each setting of treatment. Compliance with them should be employed to judge the quality of support given. Moreover, there is a strong need to increase well-conducted and scientifically sound researches in this setting, so to increase the quality of evidence and strengthen the existing guidelines.

Expert consensus papers (95–98), guidelines and survivorship care plans (99, 100) provide useful indicators for clinical practice, which are center-specific; tools to measure the quality of supportive care at an individual level are critically lacking. In view of the growing elderly and frail population affected by HNC and the ceiling of toxicity reached with standard treatments, clinical investigations on this broad topic are warranted.

## Author Contributions

All authors listed have made a substantial, direct and intellectual contribution to the work, and approved it for publication.

### Conflict of Interest Statement

PB: Advisory board: Merck, Sanofi, Merck Sharp & Dohme, Sun Pharma, Angelini, AstraZeneca. Conference honoraria: Bristol-Myers Squibb, Kyowa Hakko Kirin, Angelini, Roche. The remaining authors declare that the research was conducted in the absence of any commercial or financial relationships that could be construed as a potential conflict of interest.
